# Increased early mortality and morbidity after total hip arthroplasty in patients with socioeconomic disadvantage: a report from the Swedish Hip Arthroplasty Register

**DOI:** 10.1080/17453674.2019.1598710

**Published:** 2019-04-01

**Authors:** Rüdiger J Weiss, Johan Kärrholm, Ola Rolfson, Nils P Hailer

**Affiliations:** a Department of Molecular Medicine and Surgery, Section of Orthopaedics and Sports Medicine, Karolinska Institutet, Karolinska University Hospital, Stockholm;;; b Swedish Hip Arthroplasty Register, Gothenburg;;; c Department of Orthopaedics, Institute of Clinical Sciences, Sahlgrenska Academy, University of Gothenburg, Gothenburg;;; d Department of Surgical Sciences, Section of Orthopaedics, Uppsala University Hospital, Uppsala, Sweden

## Abstract

Background and purpose — Socioeconomic status is associated with the outcome of major surgery. We investigated the association of socioeconomic status with the risk of early mortality and readmissions after primary total hip arthroplasty (THA).

Patients and methods — We obtained information on income, education, immigration, and cohabiting status as well as comorbidities of 166,076 patients who underwent primary THA due to primary osteoarthritis (OA) from the Swedish Hip Arthroplasty Register, the Swedish National Inpatient Register and Statistics Sweden. Multivariable Cox regression models were fitted to estimate the adjusted risk of mortality or readmissions within 90 days after index surgery.

Results — Compared with patients on a low income, the adjusted risk of 30-day mortality was considerably lower in patients on a high income (hazard ratio [HR] 0.5, 95% confidence interval [CI] 0.3–0.7) and in those on a medium income (HR 0.7, CI 0.6–0.9). Similar risk reductions were found for the endpoint 90-day mortality. Patients with a high income had a lower adjusted risk of readmission for cardiovascular reasons than those with a low income (HR 0.7, CI 0.6–0.9), as had those with a higher level of education (adjusted HR 0.7, CI 0.6–0.9). Patients with higher socioeconomic status had a lower degree of comorbidities than socioeconomically disadvantaged patients. However, adjusting for socioeconomic confounders in multivariable models only marginally influenced the predictive ability of the models, as expressed by their area under the curve.

Interpretation — Income and level of education are strongly associated with early mortality and readmissions after primary THA, and both parameters are closely connected to health status. Since adjustment for socioeconomic confounders only marginally improved the predictive ability of multivariable regression models our findings indicate that comorbidities may under certain circumstances serve as an acceptable proxy measure of socioeconomic background.

Socioeconomic status is strongly associated with both access to and the outcome after major surgical interventions, including total joint replacements that represent one of the most common surgical interventions in developed countries (Kurtz et al. 2007, Hunt et al. 2013, Berstock et al. 2014, Katz et al. 2017).

The relationship between health and socioeconomic status is complex since socially deprived total hip arthroplasty (THA) patients have greater comorbidity and a higher prevalence of smoking compared with more affluent patients (Jenkins et al. 2009, Clement et al. 2011). Even in a European healthcare system with less pronounced social inequality, socioeconomically disadvantaged individuals are less healthy (Padyab et al. 2013). It was therefore postulated almost 2 decades ago that “performance measures should be stratified by socioeconomic position…” (Fiscella et al. 2000), but such stratification is rarely presented in studies on short-term complications after THA surgery.

Therefore we examined (1) how personal income, level of education, cohabiting, and immigration status affect early mortality and readmission rates after primary THA surgery due to OA, and (2) whether adjustment for such socioeconomic confounders substantially changes the attained risk estimates and the predictive ability of regression models. Early mortality was defined as 30- and 90-day mortality, and hospital readmissions were divided into those that occurred for cardiovascular disease, or for any reason.

## Patients and methods

### Data sources

We extracted all patients operated with a primary THA from the Swedish Hip Arthroplasty Register (SHAR) 1992–2012 but excluded patients operated for reasons other than primary OA of the hip (Figure 1, see Supplementary data). To avoid dependency issues only the first operation was accounted for in patients with bilateral THA. National coverage of the SHAR is complete and completeness of registration has been found to be stable around 96–98% (SHAR 2015, Soderman et al. 2000).

Age at index surgery was divided into 3 age groups (< 60, 60–75, and > 75 years). Socioeconomic factors including personal income and level of education were obtained from Statistics Sweden. Income was divided into low, middle, and high along tertiles, and level of education was divided into the categories low (9 years or less of school education), middle (high school level), and higher education (university level). Hospital type at the index operation was divided into university, county, and private hospitals. Immigration status was categorized as non-immigrant or immigrant (defined as a patient born outside of Sweden). Cohabiting status was categorized as living with a partner, irrespective of marital status, or living alone.

Data on medical comorbidities, dates of death, and readmissions (for cardiovascular reasons or for any reason) were obtained from the Swedish National Inpatient Register (SNPR) where data on contacts with healthcare providers are recorded together with International Classification of Diseases (ICD)- and procedural codes (Ludvigsson et al. 2011). Categories of comorbidity were computed using the Charlson Comorbidity Index (CCI) (Charlson et al. 1987, de Groot et al. 2003) by analysing registered ICD codes from the year preceding the index procedure but excluding the inpatient period during which the index procedure was performed. Comorbidity was categorized into 3 levels: low (CCI 0), moderate (CCI 1 or 2), and high (CCI > 2) (Deyo et al. 1992, Quan et al. 2011). Patients who died or emigrated during follow-up were identified through the Total Population Register (Statistics Sweden).

### Outcomes

Primary outcome was early mortality (30- and 90-day mortality), which was defined as death occurring within 30 or 90 days after the index procedure. Secondary endpoints were readmission due to cardiovascular reasons or for any reason within the first 90 days after the index procedure. Cardiovascular disease was defined as myocardial infarction, chronic heart failure, peripheral vascular disease, and/or cerebrovascular disease (Quan et al. 2011).

### Statistics

Follow-up started on the day of surgery and ended on the day of death, emigration, or censorship at December 31, 2012, whichever came first. Continuous data were described using medians and ranges; observed and expected frequencies were analysed using the chi-square test. 95% confidence intervals (CI) were used to describe estimation uncertainty.

Kaplan–Meier survival analysis was performed to estimate unadjusted survival. Cox proportional hazard models were fitted for each primary or secondary outcome at a time to calculate hazard ratios (HR) with CI, and confounders were subsequently included in multivariable regression models to calculate adjusted HR. The choice of covariates included in multivariable regression models was based on assessment of relevance and non-interference using directed acyclic graphs. The assumption of proportionality of hazards was investigated graphically and by calculating scaled Schoenfeld residuals. We used C-statistics to assess the area under the curve (AUC) and thus the ability of a given model to predict outcomes.

The observation period was divided into 3 different time periods (1992–1998, 1999–2005 and 2006–2012) to investigate temporal trends in the demography, comorbidity, surgical technique, and mortality in the investigated cohort.

The level of statistical significance was set at p < 0.05, but CIs were consistently used to assess estimation uncertainty.

### Characteristics of the study population

We identified 166,076 patients (57% females) with primary OA operated with a THA at a median age at surgery of 70 years (16–100). About half of the patients had a low level of education (47%; Table 1). Patients with a higher level of education or high income had a slightly lower level of comorbidities (Table 3).

**Table 1.** Description of the study population

Factor	Number	%
Total	166,076	
Women	93,855	57
Men	72,221	43
Median age at index surgery (range)	70 (16–100)	
Median follow-up, years (range)	7 (0–22)	
Year of surgery		
1992–1998	44,746	27
1999–2005	54,222	33
2006–2012	67,108	40
Income		
Low	57,397	35
Middle	55,151	33
High	53,528	32
Education		
Low	78,422	47
Middle	57,594	35
Higher	30,060	18
Cohabiting status		
Cohabiting	97,257	59
Non-cohabiting	68,819	41
Immigration status		
Non-immigrant	153,403	92
Immigrant	12,673	8
Charlson Comorbidity Index		
Low	139,197	84
Moderate	23,925	14
High	2,954	2
Hospital type		
County	125,801	76
Private	21,342	13
University	18,933	11
Implant fixation		
Cemented	136,554	82
Uncemented	13,233	8
Other^a^	16,289	10
Revision after index surgery		
No	157,698	95
Yes	8,378	5

a Hybrid, reversed hybrid, and resurfacing.

**Table 2.** Hazard ratio (HR) for mortality up to 30 days after THA

Factor	%	n	Crude HR (95% CI)	p-value	Adjusted HR (95% CI)	p-value
Age						
	0.1	16	Ref.		Ref.	
60–75 years	0.1	115	2.2 (1.3–3.7)	0.003	1.7 (1.0–2.8)	0.05
	0.5	264	9.3 (5.6–15.4)	< 0.001	5.1 (3.1–8.6)	< 0.001
Sex						
Female	0.2	176	Ref.		Ref.	
Male	0.3	219	1.6 (1.3–2.0))	< 0.001	1.9 (1.5–2.4)	< 0.001
Year of surgery						
1992–1998	0.4	174	Ref.		Ref.	
1999–2005	0.2	123	0.6 (0.5–0.7)	< 0.001	0.6 (0.5–0.8)	0.001
2006–2012	0.1	98	0.4 (0.3–0.5)	< 0.001	0.3 (0.3–0.4)	< 0.001
Income						
Low	0.4	213	Ref.		Ref.	
Middle	0.2	127	0.6 (0.5–0.8)	< 0.001	0.7 (0.6–0.9)	0.006
High	0.1	55	0.3 (0.2–0.4)	< 0.001	0.5 (0.3–0.7)	< 0.001
Education						
Low	0.3	261	Ref.		Ref.	
Middle	0.2	100	0.5 (0.4–0.7)	< 0.001	0.9 (0.7–1.1)	0.4
Higher	0.1	34	0.3 (0.2–0.5)	< 0.001	0.8 (0.6–1.2)	0.4
Cohabiting status						
Cohabiting	0.2	191	Ref		Ref.	
Non-cohabiting	0.3	204	1.5 (1.2–1.8)	< 0.001	1.4 (1.1–1.8)	0.001
Immigration status						
Non-immigrant	0.2	374	Ref.		Ref.	
Immigrant	0.2	21	0.7 (0.4–1.1)	0.1	0.8 (0.5–1.2)	0.3
Charlson Comorbidity Index						
Low	0.1	193	Ref.		Ref.	
Moderate	0.7	159	4.8 (3.9–5.9)	< 0.001	4.4 (3.6–5.4)	< 0.001
High	1.5	43	10.6 (7.6–14.7)	< 0.001	9.9 (7.0–13.9)	< 0.001
Hospital type						
County	0.3	314	Ref.		Ref.	
Private	0.1	25	0.5 (0.3–0.7)	< 0.001	0.9 (0.6–1.3	0.5
University	0.3	56	1.2 (0.9–1.6)	0.2	1.0 (0.8–1.4	0.9

Number of events = 395; adjusted for age, sex, year of surgery, income, education, cohabiting status, immigration status, Charlson Comorbidity Index and hospital type.

**Table 3.** Level of income and education divided by Charlson Comorbidity Index. Values are frequency (%)

Charlson Comorbidity Index			
Factor	Low	Moderate	High
Income			
Low	47,581 (83)	8,822 (15)	994 (2)
Middle	45,440 (82)	8,559 (16)	1,152 (2)
High	46,176 (86)	6,544 (12)	808 (2)
Level of education			
Low	64,849 (83)	12,098 (15)	1,475 (2)
Middle	48,442 (84)	8,119 (14)	1,033 (2)
Higher	25,906 (86)	3,708 (12)	446 (2)

Within the first 30 days after surgery 395 (0.2%) patients died, giving an unadjusted 30-day survival of 99.8% (CI 99.7–99.8). 709 (0.4%) patients died within 90 days, resulting in an unadjusted 90-day survival of 99.6% (CI 99.5–99.6). We identified 1,208 readmissions for cardiovascular reasons within 90 days (0.7% of the study population), and 27,197 (16% of the study population) were readmitted to hospital for any reason within this time frame.

### Ethics, funding, and potential conflicts of interests

Ethical approval was granted by the Regional Ethical Review Board in Gothenburg (approval number: 2013/360–13). No external funding was received. No competing interests were declared.

## Results

### Socioeconomic status and early postoperative mortality

When compared with patients in the low-income category, patients in the high-income category had about half the risk of death within 30 days after THA surgery (adjusted HR 0.5, CI 0.3–0.7), and patients in the middle-income category also had a lower adjusted risk with an HR of 0.7 (CI 0.6–0.9; Table 2). 90-day mortality showed similar risk reductions for patients in the high- and middle-income categories when compared with those in the lowest income category (Table 4, see Supplementary data). In contrast, the level of education was not associated with statistically significant variation in the adjusted risk of 30- or 90-day mortality after surgery (Tables 2 and 4, see Supplementary data). Patients who were non-cohabiting had an increased risk of both 30- (adjusted HR 1.4, CI 1.1–1.8) and 90-day (adjusted HR 1.6, CI 1.4–1.9) mortality when compared with patients living with a partner.

### Readmissions within 90 days

Patients in the high-income category had a lower risk of readmission for cardiovascular reasons than those in the low-income category (adjusted HR 0.7, CI 0.6–0.9), and a similar risk reduction was found for patients with a higher level of education (adjusted HR 0.7, CI 0.6–0.9). Patients living without a partner had an increased adjusted risk of readmission (HR 1.2, CI 1.1–1.4) compared with those living with a partner. Immigrants had a slightly increased risk of readmission for cardiovascular reasons compared with non-immigrants, but this finding failed to reach the level of statistical significance (HR 1.2, CI 1.0–1.5, p = 0.09; Table 5, see Supplementary data).

Patients with a high income (adjusted HR 0.9, CI 0.9–0.9) had a reduced risk for readmission for any reason, whereas patients living alone (adjusted HR 1.4, CI 1.3–1.4) and immigrants had an increased risk for readmission for any reason (adjusted HR 1.2, CI 1.1–1.2) (Table 6 and Figure 2, see Supplementary data).

### Access to healthcare and temporal changes in demography, comorbidity, and mortality

The majority of the patients were operated in county hospitals (76%), followed by private hospitals (13%) and university hospitals (11%). A higher than expected proportion of patients with a high income (observed count n = 11,035, expected count n = 6,879; p < 0.001 derived from chi-square test) and of those with a higher level of education (observed count n = 6,692, expected count n = 3,863; p < 0.001) were operated upon in private hospitals. Patients operated in private hospitals had a lower adjusted risk of 90-day mortality (adjusted HR 0.7, CI 0.5–1.0; Table 4, see Supplementary data) when compared with patients in county hospitals. Patients operated during 1999–2005 had a lower risk of early death compared with those operated 1992–1998, and those who had THA surgery during the latest period of 2006–2012 had the lowest risk of all sub-cohorts (Tables 2 and 4, see Supplementary data).

### Impact of adjustment for socioeconomic factors on the predictive ability of multivariable models

The regression model fitted for the endpoint 90-day mortality that included the covariates age, sex, comorbidity, income, and education had an AUC of 0.77 (CI 0.76–0.79), whereas the model without the covariates income and education had an AUC of 0.76 (CI 0.74–0.78). For the regression model fitted for the endpoint readmission for cardiovascular reasons within 90 days that included the covariates age, sex, comorbidity, income, and education we estimated an AUC of 0.74 (CI 0.73–0.76), and we attained a similar estimate of 0.73 (CI 0.72–0.75) for the model without the covariates income and education.

## Discussion

### Main findings

This study indicates that socioeconomic disadvantage is associated with an increased risk of both early postoperative mortality and early readmission after THA surgery. However, we also found that adjustment for socioeconomic status results in small changes in the attained risk estimates and only slightly improved C-statistics when modelling early mortality and readmissions after THA.

### Limitations and strengths

Our study is subject to incompleteness of data registration and registration errors. Misclassification bias is another limitation since our estimates of comorbidity and causes for readmissions are entirely based upon ICD codes registered by healthcare providers. If there are changes or errors in the coding of diagnoses, these will translate into erroneous measures of comorbidity. For instance, the comorbidity of patients analyzed in this study seems to have progressed over time (Table 7 and Supplementary data), a finding that could be related to an actual increase in the proportion of patients suffering from a higher burden of comorbidities during the last decade. On the other hand, caregiver reimbursement based on the DRG system may also have triggered a more zealous way of coding comorbidities, a phenomenon described as “physician code creep” (Seiber 2007).

We lack data on lifestyle-related confounders of our main outcome measures, such as body mass index, smoking, alcohol habits, and physical activity. Since these confounders may differ between socioeconomic strata they can profoundly influence the observed associations between socioeconomic variables and our outcome measures.

Mutually adjusted effect estimates derived from multivariable regression models are presented in our analysis. It has to be noted that the presented risk estimates for specific confounders can either represent total-effect or direct-effect estimates, and effect estimates related to the primary exposure cannot be interpreted in the same way as effect estimates of secondary risk factors (Westreich and Greenland 2013). We attempted to limit the problems associated with the presence of effect mediators and colliders by devising directed acyclic graphs prior to regression analyses, but the interdependency of socioeconomic status and comorbidity is strong and our reported effect estimates of secondary risk factors must therefore be interpreted with caution.

Strengths of our study includes the prospective data collection within 3 well-validated, population-based registers, creating high external validity. We chose to include only patients with primary OA of the hip as the reason for THA insertion, an import selection since some patients receiving THA for other reasons, such as those with a fracture of the femoral neck, are older and have a high burden of comorbidity, whereas those receiving a THA due to sequelae of childhood hip disorders are often younger and, possibly, fitter than those with OA. Taken together, by restricting our study population to patients with primary OA of the hip we avoided a large amount of residual confounding that would have been difficult to adjust for. The combined in-depth information on income, achieved level of education, and ethnicity is an additional valuable facet of our study, since this enables investigation of socioeconomic factors on a more profound level.

### Main findings related to previous literature

The overall 30- and 90-day mortality was low in our study, which is in accordance with other studies analysing early mortality after primary THA surgery for OA (Aynardi et al. 2009, Berstock et al. 2014). In our study, lower socioeconomic status was associated with increased 90-day mortality, which is also in agreement with US studies on this topic, although the odds ratio in 1 of these studies was slightly higher than in ours (Mahomed et al. 2003, Clement et al. 2011), an effect that is possibly attributable to larger disparity in income and education in the United States compared with Sweden.

It is described that socioeconomically deprived patients operated with a THA have greater comorbidity than more affluent patients, and deprived patients also have a higher prevalence of smoking, which has a profound impact on cardiovascular and respiratory comorbidity (Jenkins et al. 2009). Other studies indicate that patients with low incomes have a higher risk of acute adverse medical events after THA surgery (Agabiti et al. 2007). The association of lower socioeconomic status with higher mortality among hip fracture patients is known (Barone et al. 2009, Kristensen et al. 2017). Our finding of an association between early mortality after THA and non-cohabiting status is an expected finding when considering the fact that widowers have an increased mortality compared with married individuals, and that married persons have a longer life expectancy compared with unmarried persons (Hu and Goldman 1990, Shor et al. 2012). To have information on this parameter is a strength of our analysis; this facet of socioeconomic status is rarely reported in other studies.

We found a readmission rate for any reason of 16% within 90 days after surgery, which can be compared with a prevalence of 30-day readmissions of 7% after hip replacements in a US study (Brooke et al. 2015). A study on the risk of death, readmissions to hospital, and wound infections after elective primary THA in United States Medicare patients supports our findings (Mahomed et al. 2003).

THA patients in the higher socioeconomic strata were overrepresented in private hospitals. Some authors claim that wealthier patients have easier access to private sector care, which is associated with shorter waiting times (Agabiti et al. 2007), and this seems also to be true within the Swedish healthcare system.

The ability of the adjusted regression models to predict their respective outcomes was investigated by assessing model C-statistics, a term used to describe the AUC. We found AUC that varied between 0.7 and 0.8, which indicates a relatively good predictive capacity when compared with an investigation of 3 different comorbidity measures to predict the incidence of reoperations within 2 years after primary THA surgery, where the maximal AUC was 0.5 (Gordon et al. 2013). However, when models without adjustment for socioeconomic background variables were re-fitted we attained similar AUC to the previously fitted models with adjustment for socioeconomic background.

### Conclusion

Our findings support the notion that patients with socioeconomic disadvantage run a substantially higher risk of both early mortality and readmissions after THA surgery, even within the setting of a relatively egalitarian healthcare system. However, perhaps partly due to the fact that poor socioeconomic status is associated with a higher degree of comorbidities, adjusting for comorbidities may suffice in analyses where detailed information on socioeconomic background is lacking.

### Supplementary data

Tables 4–7 and Figures 1–2 are available as supplementary data in the online version of this article, http://dx.doi.org/10.1080/17453674.2019.1598710

The authors would like to thank all Swedish orthopaedic surgeons and secretaries who contributed data.

RJW and NPH: study design, database preparation, data analysis, manuscript drafting and editing. JK and OR: study design and final manuscript editing.

*Acta* thanks Per Kjaersgaard-Andersen and Stephan Maximilian Röhrl for help with peer review of this study.

## Supplementary Material

Supplemental Material
