# Supplementary material for: Increased early mortality and morbidity after total hip arthroplasty in patients with socioeconomic disadvantage: a report from the Swedish Hip Arthroplasty Register
Source: Acta Orthop. 2019 Apr 1;90(3):264–9. doi: 10.1080/17453674.2019.1598710 (PMC6534205; doi:10.1080/17453674.2019.1598710)
Supplement: Supplemental Material [file IORT_A_1598710_SM6333.pdf]

Supplementary data

Table 4. Hazard ratio (HR) for mortality up to 90 days after THA

Factor	%	n	Crude HR (95% CI)	p-value	Adjusted HR (95% CI)	p-value
Age						
< 60 years	0.1	29	Ref.		Ref.	
60–75 years	0.2	224	2.4 (1.6–3.5)	< 0.001	1.9 (1.3–2.8)	0.001
> 75 years	0.9	456	8.9 (6.1–12.9)	< 0.001	5.4 (3.7–7.9)	< 0.001
Sex						
Female	0.3	316	Ref.		Ref.	
Male	0.5	393	1.6 (1.4–1.9)	< 0.001	2.0 (1.7–2.3)	< 0.001
Year of surgery						
1992–1998	0.7	299	Ref.		Ref.	
1999–2005	0.4	221	0.6 (0.5–0.7)	< 0.001	0.6 (0.5–0.7)	< 0.001
2006–2012	0.3	189	0.4 (0.4–0.5)	< 0.001	0.4 (0.3–0.5)	< 0.001
Income						
Low	0.6	355	Ref.		Ref.	
Middle	0.4	242	0.7 (0.6–0.8)	< 0.001	0.8 (0.7–0.9)	0.01
High	0.2	112	0.3 (0.3–0.4)	< 0.001	0.5 (0.4–0.7)	< 0.001
Education						
Low	0.6	450	Ref.		Ref.	
Middle	0.3	187	0.6 (0.5–0.7)	< 0.001	1.0 (0.8–1.1)	0.6
Higher	0.2	72	0.4 (0.3–0.5)	< 0.001	1.0 (0.7–1.3)	0.8
Cohabiting status						
Cohabiting	0.3	326	Ref.		Ref.	
Non-cohabiting	0.6	383	1.7 (1.4–1.9)	< 0.001	1.6 (1.4–1.9)	< 0.001
Immigration status						
Non-immigrant	0.4	672	Ref.		Ref.	
Immigrant	0.3	37	0.7 (0.5–0.9)	0.02	0.8 (0.6–1.1)	0.1
Charlson Comorbidity Index						
Low	0.3	388	Ref.		Ref.	
Moderate	1.0	245	3.7 (3.2–4.3)	< 0.001	3.3 (2.8–3.9)	< 0.001
High	2.6	76	9.4 (7.3–12.0)	< 0.001	8.4 (6.5–10.9)	< 0.001
Hospital type						
County	0.5	574	Ref.		Ref.	
Private	0.2	42	0.4 (0.3–0.6)	< 0.001	0.7 (0.5–1.0)	0.05
University	0.5	93	1.1 (0.9–1.3)	0.5	1.0 (0.8–1.2)	0.5

Number of events = 709; adjusted for age, sex, year of surgery, income, education, cohabiting status, mmigration status, Charlson Comorbidity Index and hospital type.

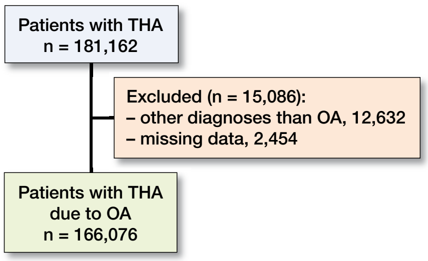

Figure 1. Patient selection.

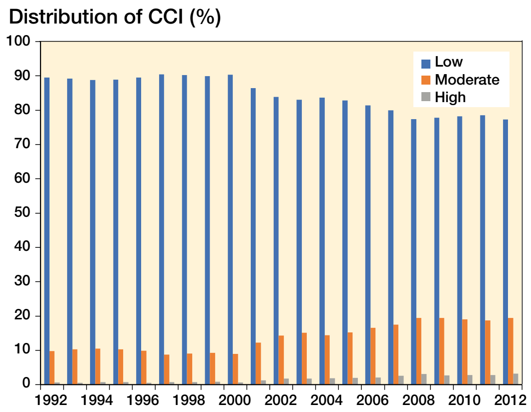

Figure 2. Distribution of Charlson Comorbidity Index (CCI) (low, moderate, and high) divided by year.

Table 5. Hazard ratio (HR) for readmissions for cardiovascular reasons up to 90 days after THA

Factor	%	n	Crude HR (95% CI)	p-value	Adjusted HR (95% CI)	p-value
Age						
< 60 years	0.1	41	Ref.		Ref.	
60–75 years	0.5	478	3.7 (2.7–5.0)	< 0.001	3.1 (2.2–4.2)	< 0.001
> 75 years	1.4	689	10.7 (7.8–14.7)	< 0.001	7.5 (5.4–10.3)	< 0.001
Sex						
Female	0.6	572	Ref.		Ref.	
Male	0.9	636	1.4 (1.3–1.6)	< 0.001	1.5 (1.4–1.8)	< 0.001
Year of surgery						
1992–1998	0.9	402	Ref.		Ref.	
1999–2005	0.8	417	0.8 (0.7–0.9)	0.003	0.8 (0.7–0.9)	0.003
2006–2012	0.6	389	0.6 (0.5–0.7)	< 0.001	0.5 (0.5–0.6)	< 0.001
Income						
Low	0.9	534	Ref.		Ref.	
Middle	0.8	439	0.8 (0.7–0.9)	0.01	1.0 (0.8–1.1)	0.5
High	0.4	235	0.5 (0.4–0.5)	< 0.001	0.7 (0.6–0.9)	0.001
Education						
Low	0.9	743	Ref.		Ref.	
Middle	0.6	347	0.6 (0.5–0.7)	< 0.001	0.9 (0.8–1.0)	0.09
Higher	0.4	118	0.4 (0.3–0.5)	< 0.001	0.7 (0.6–0.9)	< 0.001
Cohabiting status						
Cohabiting	0.7	643	Ref.		Ref.	
Non-cohabiting	0.8	565	1.3 (1.2–1.5)	< 0.001	1.2 (1.1–1.4)	0.003
Immigration status						
Non-immigrant	0.7	1112	Ref.		Ref.	
Immigrant	0.8	96	1.1 (0.9–1.3)	0.5	1.2 (1.0–1.5)	0.09
Charlson Comorbidity Index						
Low	0.5	699	Ref.		Ref.	
Moderate	1.8	429	3.8 (3.4–4.3)	< 0.001	3.4 (3.0–3.9)	< 0.001
High	2.7	80	6.2 (5.0–7.9	< 0.001	5.3 (4.2–6.7)	< 0.001
Hospital type						
County	0.8	945	Ref.		Ref.	
Private	0.5	104	0.6 (0.5–0.8)	< 0.001	1.0 (0.8–1.2)	0.9
University	0.8	159	1.2 (1.0–1.4)	0.05	1.2 (1.0–1.4)	0.1

Number of events = 1,208; adjusted for age, sex, year of surgery, income, education, cohabiting status, immigration status, Charlson Comorbidity Index and hospital type.

Table 6. Hazard ratio (HR) for readmissions for any reason up to 90 days after THA

Factor	%	n	Crude HR (95% CI)	p-value	Adjusted HR (95% CI)	p-value
Age						
< 60 years	10	24,746	Ref.		Ref.	
60–75 years	13	12,055	1.4 (1.3–1.5)	< 0.001	1.3 (1.3–1.4)	< 0.001
> 75 years	26	12,464	2.9 (2.9–3.0)	< 0.001	2.4 (2.3–2.5)	< 0.001
Sex						
Female	17	15,905	Ref.		Ref.	
Male	16	11,292	0.9 (0.9–0.9)	< 0.001	1.0 (1.0–1.1)	0.02
Year of surgery						
1992–1998	23	10,077	Ref.		Ref.	
1999–2005	16	8,671	0.7 (0.7–0.7)	< 0.001	0.7 (0.7–0.7)	< 0.001
2006–2012	13	8,449	0.5 (0.5–0.6)	< 0.001	0.5 (0.5–0.5	< 0.001
Income						
Low	19	10,858	Ref.		Ref.	
Middle	17	9,464	0.9 (0.9–0.9)	< 0.001	1.0 (1.0–1.0)	0.7
High	13	6,875	0.7 (0.6–0.7)	< 0.001	0.9 (0.9–0.9)	< 0.001
Education						
Low	19	14,763	Ref.		Ref.	
Middle	15	8,531	0.8 (0.8–0.8)	< 0.001	1.0 (1.0–1.0)	0.8
Higher	13	3,903	0.7 (0.7–0.7)	< 0.001	1.0 (0.9–1.0)	0.1
Cohabiting status						
Cohabiting	14	13,235	Ref.		Ref.	
Non-cohabiting	20	13,962	1.6 (1.5–1.6)	< 0.001	1.4 (1.3–1.4)	< 0.001
Immigration status						
Non-immigrant	16	24,880	Ref.		Ref.	
Immigrant	18	2,317	1.2 (1.1–1.2)	< 0.001	1.2 (1.1–1.2)	< 0.001
Charlson Comorbidity Index						
Low	15	20,487	Ref.		Ref.	
Moderate	24	5,694	1.7 (1.6–1.7)	< 0.001	1.7 (1.6–1.7)	< 0.001
High	34	1,016	2.6 (2.4–2.7)	< 0.001	2.5 (2.4–2.7)	< 0.001
Hospital type						
County	16	20,034	Ref.		Ref.	
Private	13	2,816	0.8 (0.8–0.9)	< 0.001	1.0 (1.0–1.0)	0.6
University	23	4,347	1.5 (1.5–1.6)	< 0.001	1.4 (1.4–1.4)	< 0.001

Number of events = 27,197; adjusted for age, sex, year of surgery, income, education, cohabiting status, immigration status, Charlson Comorbidity Index and hospital type.

Table 7. Description of the study population during 3 time periods. Values are frequency (%)

	1992–1998	1999–2005	2006–2012
Sex			
Female	25,225 (56)	30,820 (57)	37,810 (56)
Male	19,521 (44)	23,402 (43)	29,298 (44)
Age group			
< 60 years	6,488 (15)	9,411 (17)	11,525 (17)
60–75 years	24,306 (54)	28,325 (52)	37,147 (55)
> 75 years	13,952 (31)	16,486 (31)	18,436 (28)
Charlson Comorbidity Index			
Low	40,067 (89)	46,365 (85)	52,765 (79)
Moderate	4,380 (10)	7,050 (13)	12,495 (18)
High	299 (1)	807 (2)	1,848 (3)
